# The development of a robotic gynaecological surgery training curriculum and results of a delphi study

**DOI:** 10.1186/s12909-020-1979-y

**Published:** 2020-03-04

**Authors:** Aemn Ismail, Matthew Wood, Thomas Ind, Nahid Gul, Esther Moss

**Affiliations:** 1 Leicester Cancer Research Centre, University of Leicester, University Road, Leicester, LE1 7RH UK; 2 University Hospitals of Leicester, Leicester, UK; 3 Royal Marsden Hospital, London, UK; 4 Wirral University Teaching Hospital, Birkenhead, UK

**Keywords:** Minimally invasive surgery, Robotic-assisted surgery, Gynaecological surgery, Console surgeon, Surgical training

## Abstract

**Background:**

Technology for minimal access surgery is rapidly progressing in all surgical specialities including Gynaecology. As robotic surgery becomes established in increasing numbers of hospitals, there is no set curriculum for training in robotic gynaecological surgery or the assistant role in use in the UK. The purpose of this study was to determine a list of competencies that could be used as the basis of a core robotic gynaecological surgery curriculum, to explore its acceptability and the level of interest in undertaking training in robotics among obstetrics & gynaecology (O&G) trainees.

**Methods:**

A four-round Delphi study was conducted using members and associates of British & Irish Association of Robotic Gynaecological Surgeons (BIARGS). In Round 1 respondents were asked to propose standards that could be used in the curriculum. In the following three rounds, the respondents were asked to score each of the standards according to their opinion as to the importance of the standard. Items that scored a mean of 80% or above were included in the final proposed curriculum. Following this, a national survey was conducted to explore the interest among O&G trainees in undertaking a formal robotic training for the first assistant and console surgeon roles.

**Results:**

The items proposed were divided into three separate sections: competencies for a medical first assistant; competencies for a console surgeon; continued professional development for trained console surgeons. From the national survey; 109 responses were received of which 60% were interested in undertaking a formal training for the first assistant role, and 68% are expressing interest in training for the console surgeon role.

**Conclusion:**

Undertaking a Delphi exercise to determine a core gynaecological robotic training curriculum has enabled consensus to be achieved from the opinions of BIARGS members/associates. There is interest among O&G trainees at all levels of training to gain experience and develop their skills in robotic surgery by undertaking a formal training in robotic surgery at both the first assistant and console surgeon level.

## Precis

There is interest among trainees to gain robotic experience. A Delphi exercise has developed a core gynaecological robotic training curriculum with competencies for medical first assistant; console surgeon and continued professional development for trained console surgeons.

## Background

Laparoscopy has become the gold standard for the treatment of many gynecological conditions since it is associated with less postoperative pain, less blood loss, reduced analgesia requirement and shorter hospital stay [1]. With robotic surgery (RS) systems, some of the technical challenges experienced with straight-stick (SS) laparoscopy (e.g. exposure, access and wrist manipulation) can be overcome with appropriate training, due to better ergonomics, increased instrument dexterity, as well as improved visualization via 3D HD camera systems [1]. Although many procedures can be performed with straight stick laparoscopy, the learning curve for advanced procedures are reported to be much longer than with robotic surgery due to the mentioned limitations of conventional laparoscopy [2]. Prof. Jon Einarsson, the president of the American Association of Gynaecologic Laparoscopists (AAGL) stated that a 90% minimal access surgery (MAS) hysterectomy rate is a realistic goal [3]. This is supported by less than 1% abdominal hysterectomy rate in one of the UK’s National Health Service (NHS) hospitals with RS use in selected cases [4]. Over the last 15 years since its introduction; RS has steadily gained popularity in gynaecology [5], the number of RS procedures performed with the da Vinci Surgical System by Intuitive Surgical (USA) have reached over 750,000 in 2016 [6]. Gynecology is one of the specialties with the highest volume [7]. As the use of robotic surgery increases so does the demand for training and experience amongst surgeons and their trainees. Robotic-assisted surgery requires skills distinct from conventional laparoscopy or open surgery, and thus basic robotic skills training is required prior to the clinical use of the robotic systems [8]. Literature concerning training in robotic surgery states that there is a need in developing a formal training curriculum for robotic surgery [9–11]. Recently the European Association of Urology (EAU) and the Society of European Robotic Gynaecological Surgery (SERGS) have published robotic training curriculum for interested trainees [12]. The EAU Robotic Urology Section (ERUS) programme has a systematic standardised training approach and has been shown to result in trainees gaining sufficient proficiency in a shorter period of time as opposed to open feedback or a less structured surgical training [13–15]. The SERGS curriculum likewise has a very structured format with a tri-modular program including 1) Bedside console training, 2) Simulator and wet-lab training, 3) Supervised procedural training. Proficiency in robotic surgery is not currently a component of specialty training in Gynaecology in the UK and the trainees to date who have gained experience have done so as part of subspeciality training (typically gynaecological oncology) or as senior clinical fellows.

In this study; we determined a list of competencies for first assistant and console training using the Delphi technique, that could be used as the basis of a core robotic gynaecological surgery curriculum for both the console surgeon and first assistant. We also investigated whether the Obstetrics & Gynaecology (O&G) trainees in the UK have had exposure to robotics during their time in training, whether this is an area on interest to gain skills on in the future and if there is interest in developing an accreditation for a medical first assistant role.

## Methods

**Fig. 1:**
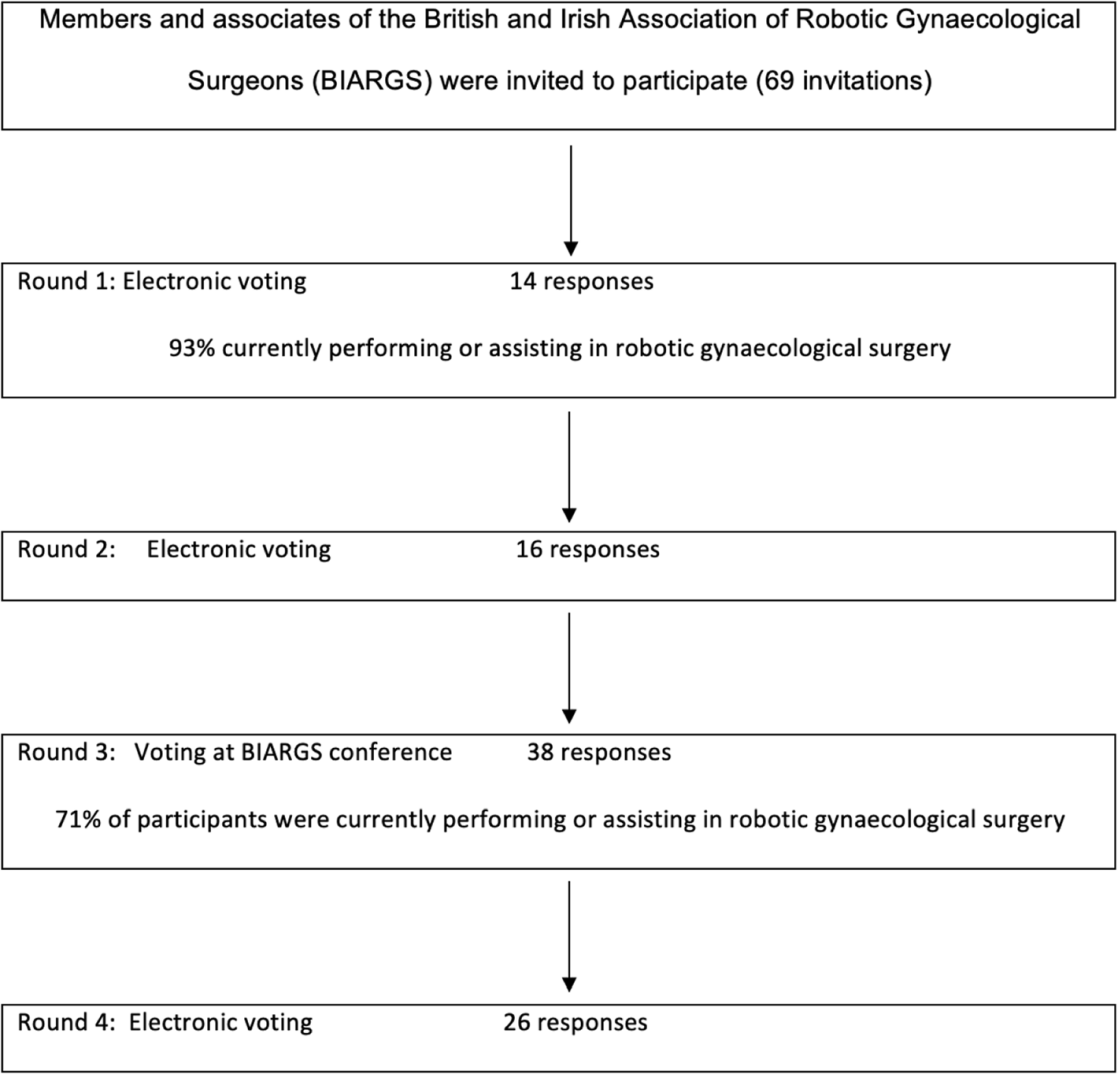
Delphi study flowchart

A four-round Delphi survey was conducted involving members and associates of the British and Irish Association of Robotic Gynaecological Surgeons (BIARGS), gynaecologists currently performing robotic surgery or undertaking learning with a view to starting a robotics program (Fig. 1). This study was undertaken by the British and Irish Association of Robotic Gynaecological Surgeons and approval was given by the executive committee. Ethical approval was not sought since the study was classified as professional development, and the participants were medical professionals, and not research as defined by the NHS Health Research Authority [16]. Participants received an invitation email contained a brief summary of the project, and contact details of the project lead in case of any questions and participation was voluntary. Written consent was not obtained but consent was implied by completion of the questionnaire. In Round 1; BIARGS members/associates were contacted electronically and asked to propose as many standards/criteria as they can that could be used in the curriculum. The responses were collected using an internet-based survey tool and divided into three separate sections: 1) Competencies for the first assistant (Medical), 2) Competencies for console surgeons, 3) Commitment to continued surgical development.

In Round 2; the BIARGS members/associates were asked to score each of the standards using a five-point Likert scale (1 = strongly disagree, 5 = strongly agree) according to their opinion as to the importance of the standard. Round 3 was conducted during the 7th Annual BIARGS conference in March 2017, Leicester, UK, where participants were asked to re-score each of the proposed competencies in light of the weighted mean scores given in Round 2. During Round 3 there was discussion amongst the members and alternative competencies were proposed, therefore a further round of scoring electronically was conducted to determine the final curriculum. Items that scored a mean of 80% or above (≥ 4 out of 5) were included in the final proposed curriculum.

Secondly, a national UK survey to O&G trainees was conducted through the Royal College of Obstetrics and Gynaecologists (RCOG) trainees’ committee. Participants were emailed a link to an electronic survey and given 10 weeks to response, one reminder email was sent after 4 weeks. Respondents were asked whether they had had exposure to robotics during their time in training, whether this was an area that they would like to gain skills in the future, and if they had interest in achieving an accreditation for the first assistant and console surgeon roles.

## Results

### The Delphi survey

In round 1 of the Delphi, 14 responses were received, and 48 standards were proposed and voted on in the following three rounds (Table 1). A curriculum of three separate sections was developed: competencies for a medical first assistant, competencies for a console surgeon, and continued professional development for trained console surgeons. In total; 16 competencies are proposed for the first assistant curriculum, 26 for the console curriculum and 4 standards for ongoing professional development. The items focused particularly on the theoretical knowledge of the robotic system, the ability to safely perform procedures/techniques, number of cases that needed to be performed in order to demonstrate competence and trouble shooting. For continued development, the importance of prospective data collective of cases and analysis/reflection of complications scored very highly. Interestingly, 58% of the participants believed that achieving a minimum of 80% (mean score of 25 responses) in simulation tests was a better standard rather than performing set number of hours on the simulator. Also, 54% of the participants were of the view that the trainee should achieve competency in performing the procedure as determined by the proctor as well as performing a minimum of 15 (calculated mean) cases under direct supervision before independent practice. 57% of participants thought that the supervising surgeon should be a trained proctor and 63% of participants believed that all surgeons should have to perform each new procedure under direct supervision until competency is achieved.

**Table 1:** Summary of the curriculum standards with their mean score given in final (Round 4) of training Delphi

Standards	Mean	Standard Deviation
Module 1: Core skills for First assistant (medical) These competencies are proposed as a curriculum for the first assistant in robotic surgery. The individual undertaking this module is assumed to have skills and experience in straight-stick laparoscopic and open surgery.		
Knowledge of the operative room setup of the robotic system	4.65	0.47
Be able to drape the robotic system	4.00	0.91
Be able to position a patient for surgery and have a knowledge of ergonomic positioning	4.69	0.46
Be able to perform the vaginal phase preparation	4.27	0.65
Have an understanding of the role and different types of uterine manipulation	4.00	0.96
Perform safe entry of trocars and port placement	4.48	0.69
Demonstrate understanding of the rationale for site port placement	4.42	0.56
Be able to dock the robotic system	4.54	0.63
Be able to trouble shoot and re-dock the robotic system	4.12	0.69
Be able to dock the robot in different positions	4.08	0.79
Be able to maintain a clear image by cleaning/changing the camera	4.75	0.43
Be able to insert, change and remove robotic instruments	4.77	0.42
Be able to adjust the arm positions to improve clearance or resolve clashing	4.46	0.63
Be able to appropriately use the assistant port	4.73	0.52
Be able to undock the robotic system	4.77	0.42
Be able to perform an emergency undocking procedure	4.77	0.42
Module 2: Core skills for Console surgeon It is assumed that the individual undertaking this module should be experienced in gynaecology with in-depth knowledge of abdominal/pelvic anatomy, be able to care for surgically unwell patients and appropriately manage intra-operative complications e.g. bowel/bladder injuries. It is assumed that the individual is able to perform the intended procedure by open surgery.		
Completion of the online robotic system theoretical training package	4.69	0.60
Awareness of the fundamentals of the robotic system components and instrumentation	4.73	0.52
Awareness of other surgical routes/modalities, and the benefits/potential complications with robotic surgery	4.69	0.53
Be able to adjust the surgical robot’s settings	4.69	0.53
Knowledge of different docking positions and the indications	4.88	0.31
Understanding of the use of electrodiathermy in robotic surgery and its potential complications	4.96	0.19
Have undertaken simulation training on a robotic simulator/trainer	4.19	1.07
Be able to turn on and calibrate the robotic system	4.04	0.59
Completion of 15^a^ cases of supervised training	3.08^b^	1.17
Be able to perform a final review of the operative set up	4.38	0.78
Be able to respond to system errors	4.73	0.52
Demonstrate camera control and set up visual field	4.85	0.36
Demonstrate clutching of the robotic instruments	4.85	0.36
Demonstrate multi-arm control of the robotic instruments	4.81	0.39
Demonstrate hand-eye instrument coordination	4.85	0.36
Demonstrate wrist articulation	4.81	0.39
Demonstrate atraumatic tissue handling	4.88	0.31
Maintain safety of operative field	4.88	0.31
Demonstrate blunt dissection with the robotic system	4.73	0.44
Demonstrate micro-dissection with the robotic system	4.46	0.57
Demonstrate safe tissue cutting with the robotic system	4.85	0.36
Demonstrate needle driving with the robotic system	4.81	0.48
Demonstrate suture handling with the robotic system	4.85	0.36
Demonstrate knot tying with the robotic system	4.77	0.42
Demonstrate continuous suturing with the robotic system	4.73	0.44
Have undertaken case observation of experienced surgeons performing robotic cases	4.42	0.74
Module 3: Commitment to continued surgical development These requirements have been proposed for surgeons who have completed their training in robotic surgery.		
Prospective audit of all robotic cases	4.46	0.57
Demonstrate analysis and reflection of complications associated with robotic surgery	4.62	0.56
Perform a minimum 25^c^ robotic cases per year	4.19	1.14
Attend emergency drill training with the robotic surgery team annually	4.31	0.72

^a^“15 cases” is the calculated mean from 25 responses

^b^This item is included despite scoring less than 80%. It scored low in view of the initially proposed number of cases to be performed under supervision (30 cases), but overall members felt that this is a very important item to be included but with less number of cases, final consensus was 15 cases

^c^mean of 26 responses

### The national survey

In total, 109 responses were received out of a total of 1795 O&G UK trainees at the time of conducting this survey. Seventy-five of the 109 (69%) respondents were at a Specialty Trainee year 3 (ST3) level or above (Table 2). None of the trainees had any experience as a console surgeon, and 62% (*n* = 63/102) of the trainees never had any robotic experience (Table 3). Overall; 66% (*n* = 64/97) felt that all trainees should have the opportunity to watch a robotic case, and 60% (*n* = 51/86) were interested in undertaking formal training for the first assistant role, whereas 68% (*n* = 59/87) were willing to consider training for the console surgeon role. The majority of the trainees thought that the robotic training should be achieved by being incorporated as a discretionary module into either the advanced laparoscopic surgery Advanced Training Skills Module (ATSM) or into Subspecialty training (43% (*n* = 47/108) and 47% (*n* = 51/108) respectively), but not as a part of the intermediate core competencies, 87% (*n* = 94/108) voted against this option.

**Table 2:** Demographics of the participants (ST = specialty training, CCT = Certificate of Completion of Training)

Participants’ training level	n (%)
ST1–2 or equivalent	34 (31.2)
ST3 or equivalent	20 (18.3)
ST4 or equivalent	12 (11.0)
ST5 or equivalent	14 (12.8)
ST6 or equivalent	16 (14.7)
ST7 or equivalent	11 (10.0)
Post CCT	2 (1.9)

**Table 3:** O&G trainees’ Robotic Gynaecological surgery experience in the UK

	None % (n)	1% (n)	2–5% (n)	6–10% (n)	11–20% (n)	> 20% (n)	Total responses
Observed	61.76 (63)	9.80 (10)	20.59 (21)	2.94 (3)	1.96 (2)	2.94 (3)	102
Second assistant	70.79 (63)	4.49 (4)	19.10 (17)	3.37 (3)	0.00 (0)	2.25 (2)	89
First assistant	86.59 (71)	1.22 (1)	3.66 (3)	1.22 (1)	1.22 (1)	6.10 (5)	82
Console surgeon	100.00 (82)	0.00 (0)	0.00 (0)	0.00 (0)	0.00 (0)	0.00 (0)	82

## Discussion

Royal College of Obstetricians and Gynaecologists (RCOG) are currently reviewing a training curriculum for the speciality to include “Capabilities in Practice” CiP. BIARGS has proposed a robotic training curriculum with similar four competency levels. Level 1 observed competencies (Second assistant for ST1 and ST2), Level 2 under direct supervision (First assistant for ST3, ST4 and ST5), Level 3 indirect supervision (Robotic ATSM), Level 4 unsupervised independent competencies (Robotic ATSM or Fellowship). Although not all Trusts in UK provide robotic surgery at present, the trainees rotate within each region and the majority of regions have at least one centre offering robotic surgery. Therefore, at present the proposal of including robotic training in the core surgical gynaecological module of RCOG curriculum will be optional. However, this will provide recognition of training for trainees who are working in robotic theatres as part of their rotations and give them platform to progress their training to the next level if there was an opportunity and they wished to do so.

In our Delphi study and trainees’ survey, we have also determined the acceptance of robotic training for both first assistance and console surgeons’ roles among trainees and their views on the feasibility of incorporating such training into the current O&G curriculum.

The Dutch Health Care Inspectorate (IGZ) stated in its published report “*Insufficiently prepared introduction of robotic surgery*” with regards to RS training that “50% of the hospitals had insufficient criteria for the surgeon’s competence before starting with robotic surgery” [17]. This highlights the need for a competence-based training curriculum in RS. Schreuder et al., concluded in a systematic review “*Training and learning RS, time for a more structured approach*” that “Robotic surgical training consists of system training and procedural training. System training should be formally organised and should be competence based, instead of time based. Procedural training should be organised in a stepwise approach with objective assessment of each step” [18]. This Delphi exercise has designed a competency-based training curriculum for the system aspect of the robotic training. However; we recognize that the procedural RS training should be achieved in a proficiency-based progression approach using instruments such as Objective Structured Assessment of Technical Skills (OSATS) for each step to capture and monitor progress.

The curriculum was designed as a competency-based curriculum (Additional file 1), which is a recognised method by the RCOG and the General Medical Council (GMC) for training that all UK O&G trainers and trainees have experience. The proposal submitted to RCOG was updated with the potential new changes to the national curriculum. There is emphasis on NOTTS (Non-Technical Skills for Surgeons) as human factor, situational awareness and communication skills are of significant importance in robotic theatre. In our study; each competence can typically be achieved in three different levels: Level 1 - observed by the trainee; Level 2 - performed by the trainee with direct supervision; Level 3 - performed unsupervised (supervisor not in room). The society of European robotic gynaecological surgery (SERGS) has developed a pilot curriculum for console training, which is considered the first standardized training programme for robotic use in gynaecological surgery [12], however the volume of training proposed is substantial, with a multi-step scheme with three key components (1) E-learning and bedside console training, (2) Training on simulators, and (3) supervised procedural training [13]. There is no doubt that such a training program is extremely comprehensive however it would be very difficult to undertake unless in a dedicated fellowship position, due to time constraints during the training, simulators, wet-lab experience and availability of virtual simulators. Open feedback together with less structured surgical training, which is used in the SERGS programme, as opposed to systematic structured competency-based curriculum, as demonstrated in our study, may result in a longer training period to gain the required competencies [7, 12]. For experienced surgeons who are adding robotic surgery to their skill set with peer mentor/proctorship a competency-based curriculum may be more appropriate, since they may be further along the learning curve due to their overall surgical experience. As fellows in the SERGS pilot curriculum request for more practical training, especially under supervision of an expert mentor [12], the BIARGS curriculum has included a minimum of 15 cases per procedure to be performed under direct supervision in order to achieve competency as a robotic console surgeon.

It recognised from the responses to the national survey that time constraints during the training and the lack of availability of the robotic systems for gynaecological use in the majority of NHS trusts are the two main contributing factors in limiting the trainees’ ability to gain robotic experience. Nearly half of trainees who had experience as a first assistant in robotic surgery had assisted in more than 20 cases, indicating that where trainees are attached to a robotic team there may be the opportunity to gain experience as a first assistant. Undertaking the proposed curriculum in the first assistant role would have enabled them to translate their time and experience as first assistant into objective training, possibly with a view of undertaking further training later on in their career as a console surgeon. Although there was interest amongst trainees for undertaking training in robotics, it was acknowledged that this would not be possible to be a mandatory part of the current 7 year specialty O&G training program, and it should only be incorporated as either a discretionary module or a robotic ATSM.

The limitations of this study are primarily related to the limited number of participants in the national survey. Reasons for this could be due either to incorrect contact details resulting in them not receiving the survey or a lack of interest in robotic surgery.

## Conclusion

There is interest among trainees at all levels to gain experience and develop their skills in robotic surgery by undertaking a formal training in robotic surgery at both the first assistant and console surgeon level, it was felt that these competencies should be incorporated as a discretionary module within relevant ATSMs or sub specialty training. Undertaking a Delphi exercise to determine this core gynaecological robotic training curriculum has enabled consensus to be achieved from the opinions of BIARGS members/associates. These results will also be used in the future discussions with the RCOG in order to incorporate robotic experience into the core training curriculum.

## Supplementary information

**Additional file 1.** The Robotic Gynaecological Surgery Training Curriculum

## Data Availability

The datasets used and/or analysed during the current study are available from the corresponding author on reasonable request.

## Abbreviations

AAGL: The American Association of Gynaecologic Laparoscopists
ATSM: Advanced Training Skills Module
BIARGS: British & Irish Association of Robotic Gynaecological Surgeons
CCT: Certificate of Completion of Training
EAU: The European Association of Urology
ERUS: The EAU Robotic Urology Section
GMC: General Medical Council
IGZ: The Dutch Health Care Inspectorate
MAS: Minimal Access Surgery
NHS: National Health Service
NOTTS: Non-Technical Skills for Surgeons
O&G: Obstetrics & Gynaecology
OSATS: Objective Structured Assessment of Technical Skills
RCOG: Royal College of Obstetricians and Gynaecologists
RS: Robotic Surgery
SERGS: The Society of European Robotic Gynaecological Surgery
SS: Straight-Stick laparoscopy
ST: Specialty Trainee
UK: United Kingdom
