# Supplementary material for: The development of a robotic gynaecological surgery training curriculum and results of a delphi study
Source: BMC Med Educ. 2020 Mar 4;20:66. doi: 10.1186/s12909-020-1979-y (PMC7057472; doi:10.1186/s12909-020-1979-y)
Supplement: Supplementary file 1 — Additional file 1. The Robotic Gynaecological Surgery Training Curriculum [file 12909_2020_1979_MOESM1_ESM.docx]

**Additional file 1:**

**The Robotic Gynaecological Surgery Training Curriculum**

**Module 1: Core skills for First assistant (medical)**

The individual undertaking this module is assumed to have skills and experience in straight-stick laparoscopic and open surgery.

| Competency | Competence level | | |
| --- | --- | --- | --- |
|  | Level 1 | Level 2 | Level 3 |
| Knowledge of the operative room setup of the robotic system |  |  |  |
| Draping the robotic system |  |  |  |
| Patient positioning for surgery and have a knowledge of ergonomic positioning |  |  |  |
| Vaginal phase preparation |  |  |  |
| Understanding of the role and different types of uterine manipulation |  |  |  |
| Safe entry of trocars and port placement |  |  |  |
| Understanding of the rationale for site port placement |  |  |  |
| Docking the robotic system |  |  |  |
| Trouble shoot and re-dock the robotic system |  |  |  |
| docking the robot in different positions |  |  |  |
| Maintain a clear image by cleaning/changing the camera |  |  |  |
| Insertion, change and removal of robotic instruments |  |  |  |
| Adjusting the arm positions to improve clearance or resolve clashing |  |  |  |
| Appropriately using the assistant port |  |  |  |
| Undocking the robotic system |  |  |  |
| Emergency undocking procedure |  |  |  |

**Module 2: Core skills for Console surgeon**

It is assumed that the individual undertaking this module should be experienced in gynaecology with in-depth knowledge of abdominal/pelvic anatomy, be able to care for surgically unwell patients and appropriately manage intra-operative complications e.g. bowel/bladder injuries. It is assumed that the individual is able to perform the intended procedure by open surgery.

| Competency | Competence level | | |
| --- | --- | --- | --- |
|  | Level 1 | Level 2 | Level 3 |
| Completion of the online robotic system theoretical training package |  |  |  |
| Awareness of the fundamentals of the robotic system components and instrumentation |  |  |  |
| Awareness of other surgical routes/modalities, and the benefits/potential complications with robotic surgery |  |  |  |
| Knowledge of different docking positions and the indications |  |  |  |
| Adjust the surgical robot's settings |  |  |  |
| Understanding of the use of electrodiathermy in robotic surgery and its potential complications |  |  |  |
| Have undertaken simulation training on a robotic simulator/trainer |  |  |  |
| Performing of a minimum of 15 cases of supervised training |  |  |  |
| Turn on and calibrate the robotic system |  |  |  |
| Perform a final review of the operative set up |  |  |  |
| Respond to system errors |  |  |  |
| Demonstrate camera control and set up visual field |  |  |  |
| Demonstrate clutching of the robotic instruments |  |  |  |
| Demonstrate multi-arm control of the robotic instruments |  |  |  |
| Demonstrate hand-eye instrument coordination |  |  |  |
| Demonstrate wrist articulation |  |  |  |
| Demonstrate continuous suturing with the robotic system |  |  |  |
| Demonstrate atraumatic tissue handling |  |  |  |
| Maintain safety of operative field |  |  |  |
| Demonstrate blunt dissection with the robotic system |  |  |  |
| Demonstrate micro-dissection with the robotic system |  |  |  |
| Demonstrate safe tissue cutting with the robotic system |  |  |  |
| Demonstrate needle driving with the robotic system |  |  |  |
| Demonstrate suture handling with the robotic system |  |  |  |
| Demonstrate knot tying with the robotic system |  |  |  |
| Have undertaken case observation of experienced surgeons performing robotic cases |  |  |  |

**Module 3: Commitment to continued surgical development**

These requirements have been proposed for surgeons who have completed their training in robotic surgery.

| Competency |
| --- |
| Prospective audit of all robotic cases |
| Demonstrate analysis and reflection of complications associated with robotic surgery |
| Perform a minimum 25 robotic cases per year |
| Attend emergency drill training with the robotic surgery team annually |
